# Estimating the threat of AI-agent responding across online survey platforms

**DOI:** 10.1073/pnas.2606460123

**Published:** 2026-10-15

**Authors:** Grace Zhang, Robert Walatka, Stephanie Y. Chen, Alexander K. Moore, Oleg Urminsky, Kianté Fernandez, Andrea Low, Jonathan E. Bogard, Craig R. Fox

**Affiliations:** ^a^ Marketing, University of Chicago Booth School of Business, Chicago, IL 60637; ^b^ Behavioral Science, University of Chicago Booth School of Business, Chicago, IL 60637; ^c^ Marketing, London Business School, London NW1 4SA, United Kingdom; ^d^ Marketing, University of Illinois Chicago College of Business Administration, Chicago, IL 60607; ^e^ Department of Psychology, University of California, Los Angeles, CA 90095; ^f^ Behavioral Decision Making Area, Behavioral Decision Making Area, Anderson School of Management, University of California, Los Angeles, CA 90095; ^g^ Organizational Behavior, Olin Business School, Organizational Behavior, Olin Business School, Washington University in St. Louis, St. Louis, MO 63130

**Keywords:** surveys, large language models, survey quality, bot detection, agentic AI

## Abstract

Recent research and advances in LLMs have led to widespread concern that AI agents could pose as human online survey-takers. However, it remains unclear how prevalent AI agents are on survey research platforms and how to effectively detect AI agents. We validated a series of AI detection tests that effectively separated verified-human participants from both prompted commercial and purpose-built AI agents. Using these tests, we collected surveys on seven online platforms and find wide variation in rates of respondents failing AI tests, ranging from 6 to 41% across platforms (compared to a 2.4% false-positive rate for in-person human participants). We further demonstrate that undetected AI agents can impact the results of online surveys. Our findings suggest that while there is an urgent need for AI detection tests and consistent, systematic monitoring of data quality on online platforms, some platforms currently appear to provide data with a low rate of AI agents.

Online data collection platforms are a key resource for researchers studying human thinking across many fields. Rapid advances in LLM-based agentic AI threaten the validity of these tools, due to the risk of a flood of AI agents posing as humans (1). Westwood (2) illustrated this threat by designing an AI agent that produced coherent survey responses, passing his attention check and AI detection tests. This demonstration of possible AI contamination has raised widespread concern (3, 4), but it is unclear how prevalent such agents currently are on online survey platforms. Calibrating the prevalence using validated AI detection tests is crucial for assessing the validity of research using recently collected online data and for researchers selecting platforms and AI detection strategies. Westwood and Frederick (5) provided an initial audit of one platform using typing speed and pasting behavior to detect AI. We provide an extensive and transparent examination of seven online platforms, testing a battery of 12 AI detection tests with both widely available and purpose-built AI agents, and, importantly, estimating false-positive rates of AI checks using in-person human participants. Data and materials can be found at: https://osf.io/x7jkh/.

## Method

We conducted a survey that included 12 AI detection tests and collected data from in-person verified-human participants, three commercially available AI agents (ChatGPT Atlas, Perplexity Comet, and Manus), six adversarial purpose-built AI agents, one highly customized adversarial agent, and seven online survey platforms [Prolific N = 200, CloudResearch (CR) Connect N = 205, CintTheorem N = 76, Verasight N = 105, Qualtrics Panel N = 102, Clickworker N = 105, CloudResearch (CR) Standard/MTurk N = 100]. See the *SI Appendix, Extended Methods*.

**Fig. 1.**
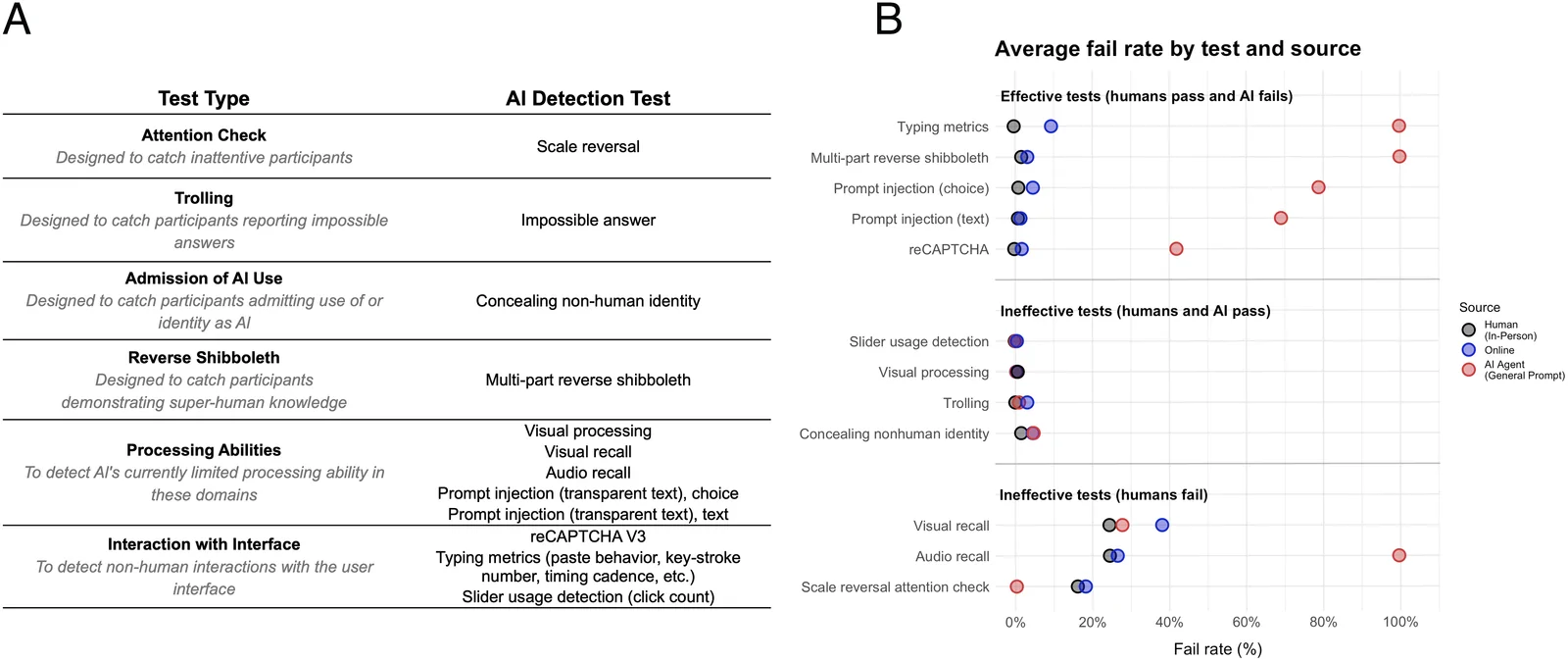
List of 12 AI detection tests used in survey (*A*) and failure rate by data source (*B*).

The survey included five types of tests used by Westwood (2): Attention check, trolling, reverse shibboleth, admission of AI use, and tests that require processing audio/visual information. We also included prompt injection tests (1) not used by Westwood (2)—instructions in transparent text (readable by AI agents only) to give a specific (unlikely) response. Finally, we included three tests designed to detect nonhuman interactions with the user interface [enhanced relative to Westwood (2) and Westwood and Frederick (5)]: reCAPTCHA v3, slider usage, and typing metrics. Our typing metrics differ from Westwood and Frederick’s (5), flagging responses as AI if they exhibited unusually fast or uniform typing cadence, unusually few keystrokes, or patterns of pasting identified as inconsistent with human behavior (but allowing those consistent with autocomplete usage). See Fig. 1*A*.

For validation, we collected responses in person (N = 211) to identify tests with low false-positive rates (<2%) among verified human participants. To examine existing AI-agent performance, we designed simple instructions (<150 words) for Atlas, Comet, and Manus on how to access and take the survey, including basic guidelines for avoiding detection as AI [to not admit being AI, report impossible events, or reveal superhuman abilities (6)]. To examine whether more customized AI agents could pass our tests, we enhanced Westwood’s (2) prompt with additional instructions relevant to the general approaches in our AI tests in both autonomous and human-assisted modes. We also conducted two adversarial challenges in which independent experts designed AI agents to complete our survey without being detected as AI, six with no knowledge of or access to our survey’s contents and one with full knowledge and the ability to test agents on our survey during development. We simulated survey responses with all of our prompts and adversarial agents. Our results only include data from runs in which the AI agent successfully completed the entire survey, as is customary for most researchers using human participants.

## Results

We first identified five effective AI detection tests with low fail rates (<2%) among verified-human respondents and substantial fail rates (>40%) among general-prompt commercial AI agents (Fig. 1 *B*, *Top*). We identified two sets of ineffective tests: those with low fail rates among both humans (<2%) and AI agents (<5%; Fig. 1 *B*, *Middle*), and those failed by humans (>16%; Fig. 1 *B*, *Bottom*). Tests with high failure rates among verified-human participants highlight the importance of a human baseline, so that high failure rates are not misinterpreted as a high prevalence of AI agents but rather as a test that is challenging for humans and therefore uninformative.

We used the five most effective tests as characterized above to estimate the prevalence of AI agents on common online platforms, classifying responses that failed any test as potential AI. Only 2.4% of our verified-human sample but 100% of our general-prompt AI agents failed this criterion. The online platforms varied greatly in the AI-test failure rate (Table 1, rightmost column), ranging from 6.0% (Prolific, comparable to verified humans) to 41.0% (CR Standard/MTurk). In contrast with verified-human surveys conducted in person by research assistants, online platform data could include noncompliant human-participant behavior (e.g., looking up answers, cutting and pasting text) that could have resulted in failing AI tests.

**Table 1.** Cumulative percent of respondents failing the effective tests

		Failed most effective test	Failed any of the top two effective tests	Failed any of the top three effective tests	Failed any of the top four effective tests	Failed any of the top five effective tests
	Detection test added	Typing metrics	+Multipart reverse shibboleth	+Prompt injection (choice)	+Prompt injection (text)	+reCAPTCHA
	Human (in-person)	0.0%	1.4%	1.9%	2.4%	2.4%^a^
Online	Prolific	1.5%	2.5%	6.0%	6.0%	6.0%^ab^
	Verasight	3.8%	3.8%	7.6%	7.6%	8.6%^bc^
	CR Connect	5.4%	6.9%	10.3%	10.3%	10.8%^bc^
	Cint Theorem	7.9%	7.9%	10.5%	10.5%	11.8%^bcd^
	Clickworker	9.5%	10.5%	14.3%	14.3%	14.3%^cd^
	Qualtrics	11.8%	13.7%	18.6%	18.6%	19.6%^d^
	CR MTurk^*^	37.0%	38.0%	38.0%	38.0%	41.0%^e^
Commercial AI agents	AI agents (general prompt)	100.0%	100.0%	100.0%	100.0%	100.0%
	AI agents, autonomous (enhanced prompt)	100.0%	100.0%	100.0%	100.0%	100.0%
	Atlas, human assisted (enhanced prompt)	63.3%	63.3%	66.7%	66.7%	66.7%
	Comet, human assisted (enhanced prompt)	100.0%	100.0%	100.0%	100.0%	100.0%
	Manus, human assisted (enhanced prompt)	93.3%	93.3%	93.3%	93.3%	96.7%
Adversarial agents	Adversarial w/o knowledge	100.0%	100.0%	100.0%	100.0%	100.0%
	Adversarial w/ knowledge^†^	0.0%	0.0%	0.0%	0.0%	N/A

Notes. Percentages in the rightmost column with different superscripts differ at the 0.05 level by a two-sample test of proportions. Only Human and Online samples were compared.

^*^MTurk announced that it was no longer accepting new customers starting at the end of July 2026.

^†^The agent designer in the adversarial challenge with knowledge did not attempt to evade the reCAPTCHA v3 as not to violate the terms and condition of web browsers.

Table 1 reports the cumulative ability of our tests to detect AI; that is, the proportion of responses flagged as potential AI, starting with the most effective test and then successively adding tests, in declining order of efficacy. In the online platform data, additional tests generally screened out more participants, highlighting the insufficiency of any single test and the benefit of including multiple, minimally correlated tests.

Additional results from the agents using our enhanced Westwood prompt or designed for our adversarial challenges help us assess whether the low failure rates on some platforms could be due to more sophisticated AI agents, like those described by Westwood (2), going undetected. The agents designed for our adversarial challenge (without knowledge of the specific tests used) and the enhanced-prompt AI agents were detected 100% of the time (result replicated using an open-source agent framework that avoids possible commercial-agent guardrails, see *SI Appendix*), with the exception of human-assisted Manus and Atlas (96.7% and 66.7% fail rate, potentially reduced by a survey design confound, see *SI Appendix*).

The adversarial agent designed with Claude Code, full knowledge of our tests, and targeted tools to help it evade AI tests represented a worst-case scenario. After iterative testing and refinement, the final agent was never detected. However, it was both slow (average time of 22.5 min vs. 8.2 for humans) and very costly ($6.23 vs. the $1 participant compensation) to run.

Finally, we demonstrate that presumed AI responses can shift survey results. At the end of the survey, participants rated the ethicality of using AI to complete online surveys. Average ethicality ratings from responses failing AI tests were significantly higher than those from respondents who passed (*M*_fail_ = 2.47, *M*_pass_ = 1.04, *t*(152.44) = 7.23, *P* < 0.001), suggesting that failed responses inflated the ratings of AI-use acceptability. Accordingly, the average ethicality rating for CR Standard/MTurk (*M* = 2.33) was significantly higher than that of our verified-human sample (*M* = 0.86, *t*(137.02) = −6.25, *P* < 0.001). However, after excluding responses flagged as potential AI, the ratings were no longer significantly different across the two groups (*M_CR_* = 1.22, *M_human_* = 0.85, *t*(78.67) = −1.50, *P* = 0.14).

## Discussion

Our tests successfully detected behavior inconsistent with human respondents, with prevalence that varied greatly across online survey platforms. Our results suggest that some online platforms may currently be relatively effective in screening for AI agents, providing likely-human data. By contrast, data from higher-failure-rate platforms raises concerns about validity, particularly if collected without effective researcher-implemented AI tests. Of course, our estimates could reflect a lower bound on AI-agent prevalence if more sophisticated agents went undetected by our tests. We think this is unlikely because adversarial agents designed without knowledge of our specific tests were fully detected and the “worst-case” adversarial agent designed with full information was both too slow and too costly to earn money completing surveys. In addition, we independently validated that the platforms with low fail rates [e.g., Prolific (7)] use enrollment procedures that seem to preclude launching many agents at scale. This said, our tests may not detect when humans augment their own answers with LLMs rather than more fully delegating to an AI agent, which is a separate challenge for data collection.

Given how rapidly AI tools advance, validity of a given platform’s data may change over time and therefore new AI tests (8) will need to be developed using transparent and reproducible methods that involve human-subjects calibration. The results of our adversarial challenges suggest that knowledge of the specific AI detection tests used is particularly critical for designing agents that can successfully evade detection. Thus, researchers should vary the tests that they use and continue to develop new ones so that those trying to use AI agents to complete surveys cannot anticipate them.

Preserving valid online data collection as a resource will likely require both: 1) that online platforms commit to investing in ongoing human verification and AI detection, and 2) ongoing independent verification of platform data validity by the research community, for which we have developed a template for here. These tests may also be combined with participant-facing interventions aimed at decreasing, rather than detecting, AI use (9). Unfortunately, this may require higher online data collection costs in the future.

AI agents taking online surveys is a specific instance of a much broader societal challenge arising from agentic AI posing as humans. AI detection involves fundamental questions about which abilities may remain uniquely human (e.g., coherent language, reasoning) as LLMs rapidly advance (10–12). Our set of effective tests did not assume lower ability of AI (vs. humans), but instead capitalized on the fact that AI completes tasks differently than humans—notably, different survey–interface interactions (typing metrics) and enhanced information processing (prompt injection and reverse shibboleth). Thus, a greater understanding of which AI detection tests are and are not effective may not only facilitate online data collection but also inform the more general debate about how AI both succeeds and fails in emulating human behavior.

This research was approved by the University of Chicago’s Social & Behavioral Sciences IRB (IRB20-0711). Participants were informed about minimal risks and provided written consent.

## Supplementary Material

Appendix 01 (PDF)

## Data Availability

Anonymized survey data have been deposited in OSF (13). Study materials are included in the *SI Appendix*.
